# Left Atrial Diameter and the Risk of Thromboembolism in Patients with Left Ventricular Noncompaction

**DOI:** 10.3390/jcdd9120426

**Published:** 2022-11-30

**Authors:** Wei Xu, Yanmin Yang, Jun Zhu, Jiangshan Tan, Jingyang Wang, Lulu Wang

**Affiliations:** Emergency Center, State Key Laboratory of Cardiovascular Disease of China, National Clinical Research Center of Cardiovascular Diseases, National Center for Cardiovascular Diseases, Fuwai Hospital, Chinese Academy of Medical Sciences and Peking Union Medical College, Beijing 100037, China

**Keywords:** left atrial diameter, left ventricular noncompaction, thromboembolism, intracardiac thrombi, stroke

## Abstract

Aims: Patients with left ventricular noncompaction (LVNC) are at risk of thromboembolism. The relationship between left atrial diameter (LAD), a robust predictor for thrombosis, and LVNC is unclear. The purpose of this study was to explore the effect of LAD on the thrombotic risk in LVNC. Methods: In this retrospective cohort study, 320 patients with imaging characteristics of LVNC were included for statistical analysis. The primary endpoint was a composite event of intracardiac thrombi and stroke or transient ischemic attack (TIA). The secondary endpoints were intracardiac thrombi and stroke/TIA. Results: The 320 included patients (211 [65.9%] men, median age: 45 years [interquartile range: 30–57]) were divided into LAD1 (<43 mm, *n* = 157) and LAD2 (≥43 mm, *n* = 163) groups based on the median LAD. Throughout the median follow-up of 34 months, the incidence of thromboembolism among them was 7.2%: 11 (3.4%) patients had stroke/TIA and 14 (4.4%) had intracardiac thrombi. The rate of thromboembolism in the LAD2 group was higher than that of patients in the LAD1 group (11.0% vs. 3.2%, *p* = 0.007). Kaplan–Meier survival curves suggested that a LAD ≥ 43 mm was associated with a higher risk of thromboembolism and intracardiac thrombi (log-rank test, all *p* < 0.05). After adjusting for potential risk factors, LAD ≥ 43 mm was found to be an independent risk factor for thromboembolism (*p* = 0.013) and stroke (*p* = 0.024). The area under the receiver operating characteristic curve of LAD for predicting thromboembolism reached 0.696 at 1 year, 0.635 at 2 years, and 0.660 at 3 years. Conclusions: A larger LAD was related to a higher risk of thromboembolism in patients with LVNC. The LAD may be a useful predictor for thrombotic risk stratification among such patients.

## 1. Introduction

Left ventricular noncompaction (LVNC) is a myocardial disease of which we have a limited understanding. It is characterized by two layers of myocardium (compacted and non-compacted), increased intertrabecular recesses, and myocardial trabeculations [1,2]. Its clinical manifestation is nonspecific, and its diagnosis remains controversial. With the increasing use of imaging-based diagnosis, according to the ratio of compacted to non-compacted layers, the diagnosis rate of LVNC has improved recently [2]. According to a recent study, the survival of patients with LVNC decreased by 9% compared with that of the gender- and age-matched population [3]. Heart failure, ventricular arrhythmias, and thromboembolism are regarded as typical complications in patients with LVNC [4]. Adult patients with LVNC are prone to developing thromboembolism; the incidence reportedly ranges from 5% to 38% [5]. However, risk stratification for thromboembolism in patients with LVNC remains challenging because of a lack of data. Thus, it is vital to explore predictors of thromboembolism in patients with LVNC. Left atrial diameter (LAD) is reportedly a robust predictor of thromboembolism and other cardiovascular events [6,7]. However, the direct effect of LAD on the thrombotic risk in patients with LVNC remains unclear. Hence, the objective of this study was to evaluate the impact of LAD on the risk of thromboembolism in patients with LVNC.

## 2. Methods

### 2.1. Study Design and Population

**Figure 1:**
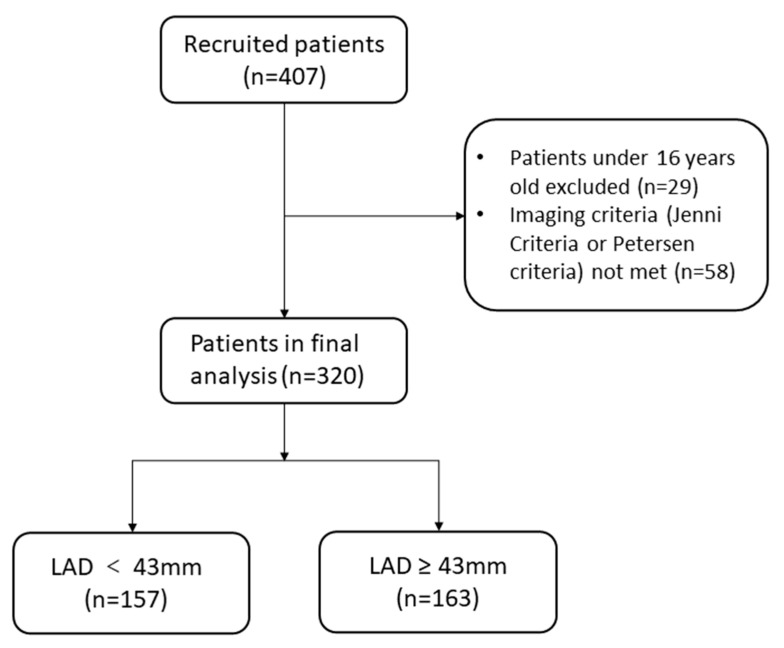
Flow chart of the study population. LAD, left atrial diameter.

The present study was a retrospective cohort study. From October 2010 to January 2021, at the Fuwai Hospital, a total of 407 patients diagnosed with LVNC were enrolled in this study. Of these, 29 who were under 16 years of age and 58 for whom diagnoses were not based on imaging criteria were excluded (Figure 1). Eventually, 320 patients met the Jenni [8] or Petersen [9] criteria (Supplementary Material—Supplementary Methods) and were included in the final analysis. The study protocol was approved by the ethics committee of Fuwai Hospital, and written informed consent was obtained from patients. The investigation conforms with the principles outlined in the Declaration of Helsinki.

### 2.2. Baseline Data Collection

We collected baseline information, including demographic data, vital signs on admission, comorbidities, and echocardiographic parameters from patients’ medical records in this study. Data on family history of cardiomyopathy, smoking status, drinking status, syncope, cardiogenic shock, and cardiopulmonary resuscitation were also collected. Comorbidities of interest were a history of heart failure, hypertension, diabetes, coronary artery disease, prior stroke or transient ischemic attack (TIA), pulmonary arterial hypertension, valvular heart disease, and atrial fibrillation. The New York Heart Association (NYHA) functional class, assessed on admission in patients with heart failure, was recorded. The CHA_2_DS_2_-VASc score was calculated based on clinical guidelines [10]. Echocardiographic parameters, including LAD, left ventricular end-diastolic diameter (LVEDD), and left ventricular ejection fraction (LVEF), were also collected.

### 2.3. Measurements

Echocardiographic parameters including LAD, LVEDD, and LVEF were evaluated on admission by using transthoracic echocardiography (Philips Medical Systems, Andover, MA, USA). The biplane Simpson’s method was used to calculate LVEF.

### 2.4. Follow-Up and Outcomes

Patients were followed up by reviewing their medical records and conducting telephonic interviews. Follow-up data collection was completed on 1 April 2022. In this study, the primary endpoint was thromboembolism, while the secondary endpoints were intracardiac thrombi and stroke/TIA. Thromboembolisms were regarded as a composite endpoint of stroke/TIA and intracardiac thrombi.

### 2.5. Statistical Analysis

Continuous variables are described as means ± SDs or medians (interquartile ranges [IQRs]) depending on whether they had a normal or skewed distribution, respectively, as verified with the Kolmogorov–Smirnov test. Thereafter, Student’s *t*-test or the Mann–Whitney U test were used to compare them, respectively. On the other hand, categorical variables are described as frequencies and percentages, and chi-square tests were used to compare them. Kaplan–Meier survival curves were plotted and compared by using log-rank tests. We also conducted univariate and multivariable Cox regression analyses to analyze the effect of variables on endpoints, calculating the hazard ratios (HRs) and a 95% confidence interval (CIs). Subgroup analyses were also performed to evaluate the relationship between LAD and thromboembolism. Moreover, time-dependent receiver operating curves (ROC) were conducted, and the area under the curve (AUC) were calculated to assess the predictive value of LAD for thrombotic risk. The predictive valuescalculated as AUC can be interpreted as negligible (AUC ≤ 0.55), small (0.56 ≤ AUC ≤ 0.63), moderate (0.64 ≤ AUC ≤ 0.70), and strong (AUC ≥ 0.71) [11]. Statistical analyses were conducted using IBM SPSS Statistics version 26.0 (IBM Corp., Armonk, NY, USA) and R version 4.0.4 (R Foundation for Statistical Computing, Vienna, Austria). All statistical analyses were two-tailed, and *p*-values < 0.05 were regarded as statistically significant.

## 3. Results

### 3.1. Baseline Characteristics

**Figure 2:**
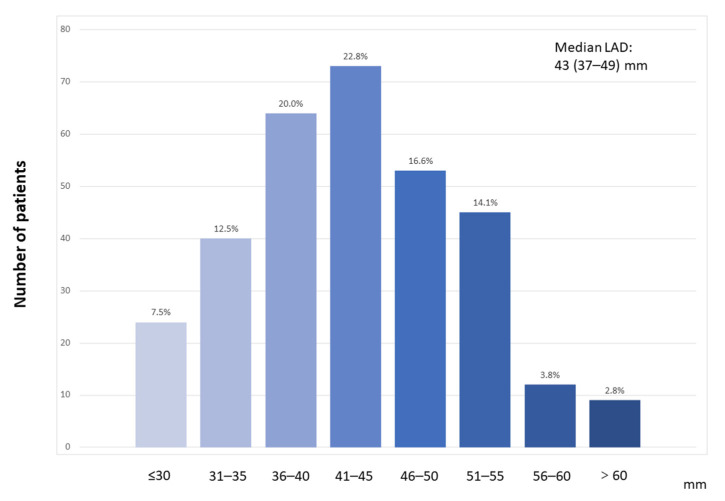
Distributions of left atrial diameter. LAD, left atrial diameter.

Figure 2 shows the distribution of patients’ LAD upon echocardiography. The median LAD was 43 (IQR, 37–49) mm. A total of 320 patients with LVNC were divided into two groups according to the median LAD: the LAD1 (LAD < 43 mm, *n* = 157) and LAD2 (LAD ≥ 43 mm, *n* = 163) groups. The baseline characteristics of patients with LVNC are described in Table 1. Among the entire study population, 211 (65.9%) patients were male, and the median age was 45 years (IQR, 30–57). In total, 19 (5.9%) patients had a family history of cardiomyopathy, and 56 (17.5%) had a history of syncope. Notably, 7 (2.2%) patients experienced cardiogenic shock, while 12 (3.8%) patients had a history of cardiopulmonary resuscitation. In total, 83.4% of patients had heart failure, 33.8% had NYHA class III heart failure, and 19.1% had NYHA class IV heart failure. In addition, 18.4% of patients had coronary artery disease, 19.4% had atrial fibrillation, and 8.8% had a history of stroke/TIA. Patients in the LAD2 group tended to be men and to have a higher BMI, higher height, lower systolic blood pressure, and higher heart rate than those in the LAD1 group (all *p* < 0.05). They were also more likely to have a history of smoking, drinking, heart failure, pulmonary arterial hypertension, valvular heart disease, and atrial fibrillation (all *p* < 0.05). The proportion of patients with NYHA class III or IV heart failure and the value of LVEDD were higher, while the LVEF level and proportion of digoxin or diuretics using were lower in the LAD2 group (all *p* < 0.05).

### 3.2. Clinical Outcomes

In this study, 320 patients with LVNC were followed up for a median of 34 months. The incidence of thromboembolism among them was 7.2% (Table 2): 11 (3.4%) patients had a stroke/TIA and 14 (4.4%) had intracardiac thrombi (Supplementary Materials—Table S1). The rates of thromboembolisms were 3.2% and 11.0% in the LAD1 and LAD2 groups, respectively (*p* = 0.007). The incidences of stroke (1.3% vs. 5.5%) and intracardiac thrombi (1.9% vs. 6.8%) were significantly higher in the LAD2 group compared with those in the LAD1 group (both *p* < 0.05).

**Figure 3:**
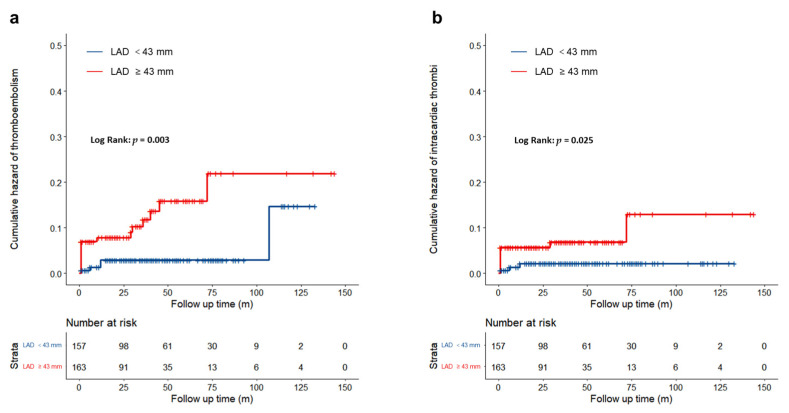
Kaplan–Meier analysis according to left atrial diameter. (**a**): Thromboembolism events; (**b**): Intracardiac thrombi.

The Kaplan–Meier survival curves (Figure 3) suggested that a LAD ≥ 43 mm was significantly associated with a higher risk of a thromboembolism (log-rank test, *p* = 0.003) (Figure 3a), and intracardiac thrombi (log-rank test, *p* = 0.025) (Figure 3b).

**Figure 4:**
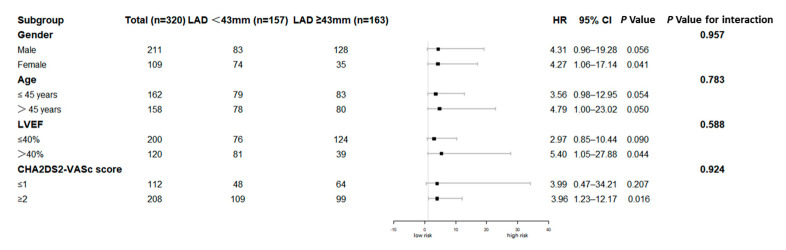
Subgroup analysis for associations between left atrial diameter and thromboembolisms in patients with left ventricular noncompaction. CI, confidence interval; HR, hazard ratio; LAD, left atrial diameter; LVEF, left ventricular ejection fraction.

As shown in Table 3, univariate Cox regression analyses in model 1 suggested that a LAD above the median (LAD ≥ 43 mm) was significantly associated with an increased risk of a thromboembolism (HR = 3.983, 95% CI = 1.474–10.763, *p* = 0.006), intracardiac thrombi (HR = 3.842, 95% CI = 1.067–13.829, *p* = 0.039), and stroke (HR = 5.017, 95% CI = 1.082–23.274, *p* = 0.039). After adjusting for age and sex in model 2, LAD above the median was statistically correlated with a higher risk of a thromboembolism (HR = 4.435, 95% CI = 1.605–12.256, *p* = 0.004), intracardiac thrombi (HR = 4.212, 95% CI = 1.132–15.673, *p* = 0.032), and stroke (HR = 5.323, 95% CI = 1.117–25.368, *p* = 0.036). When adjusted for age, sex, NYHA functional class III to class IV, prior stroke/TIA, atrial fibrillation, pulmonary arterial hypertension, LVEF < 40%, LVEDD, and anticoagulants use in model 3, a LAD ≥ 43 mm was an independent risk factor for a thromboembolism (*p* = 0.013) and stroke (*p* = 0.024). Patients with a LAD ≥ 43 mm exhibited a 4.168-fold increase in the risk of a thromboembolism (95% CI = 1.344–12.927) and a 8.654-fold increase in the risk of stroke (95% CI = 1.335–56.104) compared with patients with a LAD < 43 mm. However, the relationship between a LAD ≥ 43 mm and the risk of intracardiac thrombi was not statistically significant (HR = 3.611, 95% CI = 0.890–14.622, *p* = 0.072). In the subgroup analysis (Figure 4), patients with a LAD ≥ 43 mm consistently exhibited a higher risk of a thromboembolism than those with a LAD < 43 mm, regardless of sex, age, LVEF, or CHA_2_DS_2_-VASc score.

**Figure 5:**
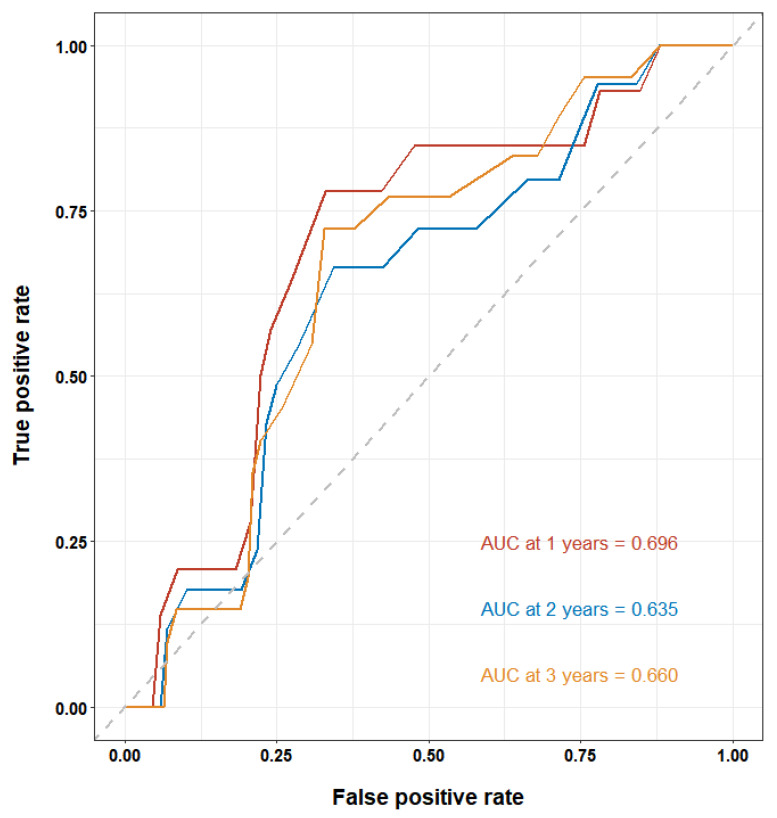
Time-dependent ROC curves of LAD for predicting thrombotic risk.

In the time-dependent ROC analysis, we assessed the predictive value of the LAD for the risk of a thromboembolism in patients with LVNC. The AUC of the LAD as predictor of a thromboembolism reached 0.696 at 1 year, 0.635 at 2 years, and 0.660 at 3 years (Figure 5), all suggesting a moderate predictive ability. The LAD cutoff value that maximized the sensitivity and specificity for predicting a thromboembolism was 43.5 mm.

## 4. Discussion

### 4.1. Main Findings

Given the low prevalence of LVNC, this study represented a relatively large cohort of patients that fulfilled the imaging-based diagnostic criteria of LVNC. In this study, 320 patients were followed up for a median of 34 months. The incidence of thromboembolism among them was 7.2%. Notably, we confirmed that a larger LAD related to an increased risk of a thromboembolism in this study sample. This relationship remained even after adjusting for potential clinical risk factors. All of the results from subgroup analyses were consistent with those of the main analyses in this study. In addition, time-dependent ROC curve analyses revealed that the predictive values of the LAD all reached a moderate predictive ability at 1 year, 2 years, and 3 years.

### 4.2. Thromboembolism in Patients with LVNC

More intertrabecular recesses and deep myocardial trabeculations are considered key anatomical structures of LVNC [1]. Generally, rough myocardial trabeculations and slow blood flow in intertrabecular recesses have been speculated to contribute to mural thrombus formation [4,12]. However, the prevalence of thromboembolisms in patients with LVNC remains unclear. Stöllberger et al. collected reports of cardiac anatomical pathologies of 37 patients with LVNC, revealing that 9 (24%) had had prior thromboembolisms and 2 (5%) had thrombi [13]. According to a review of 22 articles, the incidence of thromboembolism ranges from 5% to 38% [5]. One meta-analysis of 35 studies and 2271 patients revealed that the mean incidence of systemic thromboembolisms in patients with LVNC was 9% [12]. Consistent with previous studies, 25 patients with LVNC in our study experienced thromboembolism, with an incidence of 7.8%.

### 4.3. Left Atrial Dilation and Thrombotic Risk

In the past three decades, an association between left atrial enlargement and adverse cardiovascular events, especially stroke and systemic embolisms, has been discovered [14]. In a retrospective study, Guttmann et al. observed a positive relation between LAD and the risk of thromboembolism in patients with hypertrophic cardiomyopathy [15]. Recently, in a study of 585 patients with LVNC, Casas et al. reported that LAD was an independent risk factor for a systemic embolism [2], which is consistent with our study. However, they did not evaluate the LAD as a main objective. In our study, the incidence of thromboembolism was significantly increased in patients with a LAD above the median. However, the mechanism underlying the association remains unclear. Left atrial dilation may increase the propensity for blood stasis, which can promote thrombosis formation and embolization [14]. On the other hand, Di Tullio et al. mentioned that left atrial enlargement may be merely a risk predictor for thrombosis rather than its pathological mechanism [16]. Moreover, patients with an enlarged LAD tend to develop atrial fibrillation, according to previous studies [17], which is also considered a potential risk factor for thromboembolism [5]. However, a retrospective study, conducted in a cohort of older patients without atrial fibrillation, suggested that a larger left atrium was a robust predictor of ischemic stroke even in such patients [18]. That result indicated that the relationship between left atrial enlargement and ischemic stroke does not always involve the development of atrial fibrillation [15]. In our study, after adjusting for potential risk factors, including atrial fibrillation, a larger LAD was still significantly associated with a higher risk of thromboembolism and stroke/TIA. Our results confirmed that a LAD ≥ 43 mm is an independent risk factor for thromboembolism and stroke/TIA.

The left atrial volume index (LAVI) is a more accurate method for assessing left atrial volume, while LAD may underestimate left atrial volume [19]. However, it is difficult to measure LAVI in real-world clinical practice. In contrast, data on LAD are easier to measure and can be repeatable for most patients [20]. In this current study, we lack data on LAVI and cannot compare the prognostic value of LAVI and LAD on the thrombotic risk among patients with LVNC, directly. A Korean study that enrolled 8159 patients with atrial fibrillation demonstrated that there was no significant difference between the ability of LAVI and LAD to predict the risk of thrombosis [21]. The prognostic value of LAVI on thrombotic risk among patients with LVNC should be evaluated in future studies.

### 4.4. Clinical Implications and Future Directions

We observed that the LAD has a moderate predictive ability for the risk of a thromboembolism in such patients, according to the time-dependent ROC curves. This finding provides a new perspective on thrombotic risk stratification using the LAD in patients with LVNC. Moreover, it should be mentioned that LAD should be monitored regularly in such patients.

The protective effect of anticoagulant therapy in patients with LVNC still remains controversial [22]. Our results revealed that, in patients with LVNC who have a larger LAD, extra care is needed to reduce the risk of a thromboembolism. The addition of prophylactic anticoagulation therapy for patients with LVNC and left atrial enlargement may be needed. However, prospective, large-scale clinical trials are required to confirm this hypothesis in the future.

## 5. Limitations

Several limitations should be considered in this study. First of all, although the patients came from across China, as a single-center, observational study, potential selection biases and systematic errors might have influenced the study results. Second, as the incidences of the endpoints were relatively low in this study, the statistical robustness might have been influenced. Third, during the follow-up period, data on LAD changes and de novo atrial fibrillation was not collected in this current study; the further impact of these variables on the thrombotic risk should be evaluated in future studies.

## 6. Conclusions

Our results indicate that a larger LAD might represent a higher risk of thromboembolism in patients with LVNC. The LAD may be a useful predictor for thrombotic risk stratification in the future.

## Figures and Tables

**Table 1:** Baseline characteristics of patients with left ventricular noncompaction.

	Total	LAD1 (LAD < 43 mm)	LAD2 (LAD ≥ 43 mm)	*p*-Value
*n*	320	157	163	
Male [n (%)]	211 (65.9%)	83 (52.9%)	128 (78.5%)	<0.001
Age (y)	45 (30–57)	45 (29–57)	45 (30–57)	0.689
Height (cm)	168.8 ± 8.4	167.3 ± 8.2	170.4 ± 8.3	0.001
BMI (kg/m^2^)	23.56 ± 4.16	22.86 ± 4.00	24.22 ± 4.20	0.004
SBP (mmHg)	115 (104–128)	120 (105–130)	110 (102–123)	0.012
DBP (mmHg)	72 (65–80)	71 (67–80)	72 (65–80)	0.522
HR (beats/min)	75 (66–88)	72 (65–85)	78 (68–89)	0.023
Cardiomyopathy family history [*n* (%)]	19 (5.9%)	8 (5.1%)	11 (6.7%)	0.453
Smoking [*n* (%)]	115 (35.9%)	45 (28.7%)	70 (42.9%)	0.007
Drinking [*n* (%)]	115 (35.9%)	47 (29.9%)	68 (41.7%)	0.020
Syncope [*n* (%)]	56 (17.5%)	34 (21.7%)	22 (13.5%)	0.055
Cardiogenic shock [*n* (%)]	7 (2.2%)	5 (3.2%)	2 (1.2%)	0.231
CPR [*n* (%)]	12 (3.8%)	4 (2.5%)	8 (4.9%)	0.267
Comorbidities				
Heart failure [*n* (%)]	267 (83.4%)	114 (72.6%)	153 (93.9%)	<0.001
NYHA class [*n* (%)]				<0.001
I	25 (7.8%)	18 (11.5%)	7 (4.3%)	
II	73 (22.8%)	39 (24.8%)	34 (20.9%)	
III	108 (33.8%)	45 (28.7%)	63 (38.7%)	
IV	61 (19.1%)	12 (7.6%)	49 (30.1%)	
Coronary artery disease [*n* (%)]	59 (18.4%)	33 (21.0%)	26 (16.0%)	0.243
Pulmonary arterial hypertension [*n* (%)]	90 (28.1%)	17 (10.8%)	73 (44.8%)	<0.001
Valvular heart disease [*n* (%)]	147 (45.9%)	45 (28.7%)	102 (62.6%)	<0.001
Hypertension [*n* (%)]	84 (26.3%)	47 (29.9%)	37 (22.7%)	0.141
Diabetes mellitus [*n* (%)]	40 (12.5%)	17 (10.8%)	23 (14.1%)	0.375
Atrial fibrillation [*n* (%)]	62 (19.4%)	18 (11.5%)	44 (27.0%)	<0.001
Prior stroke/TIA [*n* (%)]	28 (8.8%)	15 (9.6%)	13 (8.0%)	0.617
CHA_2_DS_2_VASc score [*n* (%)]				0.103
≤1	112 (35.0%)	48 (30.6%)	64 (39.3%)	
≥2	208 (65.0%)	109 (69.4%)	99 (60.7%)	
Echocardiography				
LAD (mm)	43(37–49)	37 (23–40)	49 (45–53)	<0.001
LVEDD (mm)	63 (56–70)	59 (53–65)	67 (59–73)	<0.001
LVEF (%)	35 (27–52)	43 (30–57)	30 (25–40)	<0.001
LVEF < 40% [*n* (%)]	190 (59.4%)	69 (44.0%)	121 (74.2%)	<0.001
Extent of noncompaction				
Apex-only involvement [*n* (%)]	52 (17.5%)	20 (12.7%)	32 (19.6%)	0.095
Mid or basal involvement [*n* (%)]	245 (82.5%)	117 (74.5%)	128 (78.5%)	0.040
Medications in hospital				
Antiplatelets [*n* (%)]	133 (41.6%)	74 (47.1%)	59 (36.2%)	0.024
Oral anticoagulants [*n* (%)]	91 (28.4%)	37 (23.6%)	54 (33.1%)	0.079
Digoxin [*n* (%)]	141 (44.1%)	49 (31.2%)	92 (56.4%)	<0.001
ACEI/ARB [*n* (%)]	201 (62.8%)	95 (60.5%)	106 (65.0%)	0.403
ARNI [*n* (%)]	29 (9.1%)	12 (7.6%)	17 (10.4%)	0.385
Beta blockers [*n* (%)]	253 (79.1%)	127 (80.9%)	126 (77.3%)	0.430
CCBs [*n* (%)]	43 (13.4%)	18 (11.5%)	25 (15.3%)	0.310
Diuretics [*n* (%)]	266 (83.1%)	115 (73.2%)	151 (92.6%)	<0.001
Statins [*n* (%)]	101 (31.6%)	53 (33.8%)	48 (29.4%)	0.407

ACEI, angiotensin-converting enzyme inhibitor; ARB, angiotensin II receptor blocker; ARNI, angiotensin receptor neprilysin inhibitor; BMI, body mass index values; CCBs, calcium channel blockers; CPR, cardio-pulmonary resuscitation; DBP, diastolic blood pressure; LAD, left atrial diameter; LVEDD, left ventricular end diastolic diameter; LVEF, left ventricular ejection fraction; HR, heart rate; NYHA, New York Heart Association; SBP, systolic blood pressure; TIA, transient ischemic attack.

**Table 2:** Incidence of clinical events according to left atrial diameter.

Outcomes	Total (*n* = 320)	LAD < 43 mm (*n* = 157)	LAD ≥ 43 mm (*n* = 163)	*p*-Value
Thromboembolism events [*n* (%)]	23 (7.2%)	5 (3.2%)	18 (11.0%)	0.007
Intracardiac thrombi [*n* (%)]	14 (4.4%)	3 (1.9%)	11 (6.8%)	0.034
Stroke/TIA [*n* (%)]	11 (3.4%)	2 (1.3%)	9 (5.5%)	0.037

LAD, left atrial diameter; TIA, transient ischemic attack.

**Table 3:** Association between left atrial diameter ≥ 43 mm and endpoints in patients with left ventricular noncompaction.

	Model 1			Model 2			Model 3		
Outcomes	HR	95% CI	*p*-Value	HR	95% CI	*p*-Value	HR	95% CI	*p*-Value
Thromboembolism events	3.983	1.474–10.763	0.006	4.435	1.605–12.256	0.004	4.168	1.344–12.927	0.013
Intracardiac thrombi	3.842	1.067–13.829	0.039	4.212	1.132–15.673	0.032	3.611	0.890–14.662	0.072
Stroke/TIA	5.017	1.082–23.274	0.039	5.323	1.117–25.368	0.036	8.654	1.335–56.104	0.024

Model 1: Original model. Model 2: Adjusted for age and sex. Model 3: Adjusted for age, sex, NYHA functional class III to class IV, prior stroke/TIA, atrial fibrillation, pulmonary arterial hypertension, LVEF < 40%, LVEDD, anticoagulants. CI, confidence interval; HR, hazard ratio; LAD, left atrial diameter; LVNC, left ventricular noncompaction; NYHA, New York Heart Association; TIA, transient ischemic attack; LVEF, left ventricular ejection fraction; LVEDD. left ventricular end diastolic diameter.

## Data Availability

The data underlying this article cannot be shared publicly due to patient confidentiality. The data will be shared on reasonable request with the corresponding author.

## Supplementary Materials

The following supporting information can be downloaded at: https://www.mdpi.com/article/10.3390/jcdd9120426/s1, Table S1: Detailed information on intracardiac thrombi.
