# Supplementary material for: Left Atrial Diameter and the Risk of Thromboembolism in Patients with Left Ventricular Noncompaction
Source: J Cardiovasc Dev Dis. 2022 Nov 30;9(12):426. doi: 10.3390/jcdd9120426 (PMC9788599; doi:10.3390/jcdd9120426)
Supplement: Supplementary file 1 [file jcdd-09-00426-s001.zip › jcdd-2017262-supplementary.pdf]

Supplementary Material

Supplemental Methods

1. Jenni echocardiographic diagnostic criteria:

(1) Coexisting cardiac abnormalities were absent (by definition).

(2) A two layer structure was seen, with a compacted thin epicardial band and a much thicker non-compacted endocardial layer of trabecular meshwork with deep endomyocardial spaces. A maximal end systolic ratio of non-compacted to compacted layers of > 2 is diagnostic.

(3) The predominant localisation of the pathology was to mid-lateral (seven of seven patients), apical (six), and mid-inferior (seven) areas. The pathological preparations confirmed the echocardiographic findings. Concomitant regional hypokinesia was not confined to the non-compacted segments.

(4) There was colour Doppler evidence of deep perfused intertrabecular recesses.

2. Petersen criteria:

The ratio of enddiastolic noncompacted to compacted myocardium evaluating through cardiovascular magnetic resonance ≥ 2.3

Supplemental Tables

Table S1. Detailed information on intracardiac thrombi

Total	14
Left atrial appendage thrombus	4 (28.6%)
Left ventricular thrombus	10 (71.4%)
